# Proteasome Inhibitor YSY01A Abrogates Constitutive STAT3 Signaling via Down-regulation of Gp130 and JAK2 in Human A549 Lung Cancer Cells

**DOI:** 10.3389/fphar.2017.00476

**Published:** 2017-08-24

**Authors:** Wei Huang, Xia Yuan, Ting Sun, Shujie Fan, Jun Wang, Quan Zhou, Wei Guo, Fuxiang Ran, Zemei Ge, Huayu Yang, Runtao Li, Jingrong Cui

**Affiliations:** ^1^Department of Pharmacology, Institute of Basic Medical Sciences, Chinese Academy of Medical Sciences and School of Basic Medicine, Peking Union Medical College Beijing, China; ^2^State Key Laboratory of Natural and Biomimetic Drugs, School of Pharmaceutical Sciences, Peking University Beijing, China; ^3^Department of Medicinal Chemistry, School of Pharmaceutical Sciences, Peking University Beijing, China; ^4^Department of Liver Surgery, Peking Union Medical College Hospital, Chinese Academy of Medical Sciences and Peking Union Medical College Beijing, China

**Keywords:** proteasome inhibitor, YSY01A, STAT3 signaling, protein degradation, non-small cell lung carcinoma

## Abstract

Proteasome inhibition interfering with many cell signaling pathways has been extensively explored as a therapeutic strategy for cancers. Proteasome inhibitor YSY01A is a novel agent that has shown remarkable anti-tumor effects; however, its mechanisms of action are not fully understood. Here we report that YSY01A is capable of suppressing cancer cell survival by induction of apoptosis. Paradoxically, we find that YSY01A abrogates constitutive activation of STAT3 via proteasome-independent degradation of gp130 and JAK2, but not transcriptional regulation, in human A549 non-small cell lung cancer cells. The reduction in gp130 and JAK2 can be restored by co-treatment with 3-methyladenine, an early-stage autophagy lysosome and type I/III PI3K inhibitor. YSY01A also effectively inhibits cancer cell migration and lung xenograft tumor growth with little adverse effect on animals. Thus, our findings suggest that YSY01A represents a promising candidate for further development of novel anticancer therapeutics targeting the proteasome.

## Introduction

The ubiquitin-proteasome system is central for degradation of most cellular proteins that govern cell growth and differentiation, signal transduction, cell cycle regulation, and apoptosis. Protein degradation is exquisitely regulated within the cell to maintain protein homeostasis and eliminate misfolded or damaged proteins (Grigoreva et al., 2015). In cancer cells, improper regulation of the homeostatic function contributes to the development of malignant phenotype (Catalgol et al., 2012). Therefore, targeting the regulation of protein production and destruction has been a major focus of cancer research. Proteasome inhibition leads to stabilization and accumulation of its substances and consequently induces apoptosis via various cellular processes in multiple cell types, particularly in rapidly proliferating cells (Shah et al., 2002). Accordingly, the proteasome serves as an attractive target for the development of anticancer therapeutics.

In fact, various proteasome inhibitors have been identified over the past two decades (Pevzner et al., 2013). Bortezomib (also called PS-341) is the first FDA-approved proteasome inhibitor for treatment of multiple myeloma or relapsed/refractory mantle cell lymphoma (Chen et al., 2011). Numerous studies have shown that bortezomib suppresses the NF-κB regulated expression of anti-apoptotic target genes (Fahy et al., 2005; Juvekar et al., 2011; Yang et al., 2012). Meanwhile, bortezomib induces apoptosis together with autophagic formation (Lou et al., 2013; Selimovic et al., 2013). Subsequently, autophagy-mediated lysosomal degradation of key protein has also shown to contribute to cytotoxic effect of bortezomib (Fang et al., 2012). Several preclinical studies also report its ability to confer a chemo-sensitizing effect in combination with cisplatin, doxorubicin, gemcitabine, and radiation therapy (Lee et al., 2011; Ding et al., 2015; Konac et al., 2015). Bortezomib is the first proteasome inhibitor to enter clinical trials. Despite clinical success of bortezomib, toxicities and resistance have been observed. Indeed, patients who developed bortezomib resistance within an average of 1 year from the beginning of the treatment accounted for ~60% (McConkey and Zhu, 2008). The second-generation proteasome inhibitors have been also developed with different properties, such as carfilzomib, marizomib, and ixazomib (Potts et al., 2011; Offidani et al., 2014; McBride et al., 2015). In 2015, ixazomib was approved in the US for the treatment of patients with multiple myeloma who have received at least one prior therapy in combination with lenalidomide and dexamethasone. Apparently, clinical success of proteasome inhibitors have validated the proteasome as a promising therapeutic target. Nevertheless, owning to poor pharmacodynamic and pharmacokinetic properties, severe side effects and unsatisfactory efficacy in the treatment of solid tumors, it is of considerable interest to extend the benefits of proteasome inhibitors for the treatment of solid tumor malignancies.

In previous study, we identified a novel proteasome inhibitor, YSY01A, which exhibits antitumor effects *in vitro* and *in vivo* (Zhu et al., 2009). Moreover, further studies demonstrate that it modulates a variety of signaling pathways involved in the control of cell proliferation, cell cycle, apoptosis, and autophagy (Wang et al., 2014; Xue et al., 2015; Zhang et al., 2015; Huang et al., 2016b). We have shown that YSY01A suppresses survival of cisplatin-resistant ovarian cancer cells by inducing apoptosis and characterized the signaling cascades mediating these effects (Huang et al., 2016b). Specifically, YSY01A suppresses regulatory proteins favoring cell proliferation and anti-apoptosis including NF-κB and STAT3 pathways, leading to down-regulation of anti-apoptotic bcl-2. In this report, we further define a novel mechanism whereby YSY01A suppresses survival and migration of A549 non-small cell lung cancer cells by abrogating constitutively active STAT3 signaling cascade. Our study also evaluated *in-vivo* efficacy of YSY01A in A549 mouse xenograft model. Collectively, YSY01A promises a potential lead candidate for further development of anticancer therapeutics by inhibition of the proteasome.

## Materials and methods

### Chemicals and reagents

**Figure 1:**
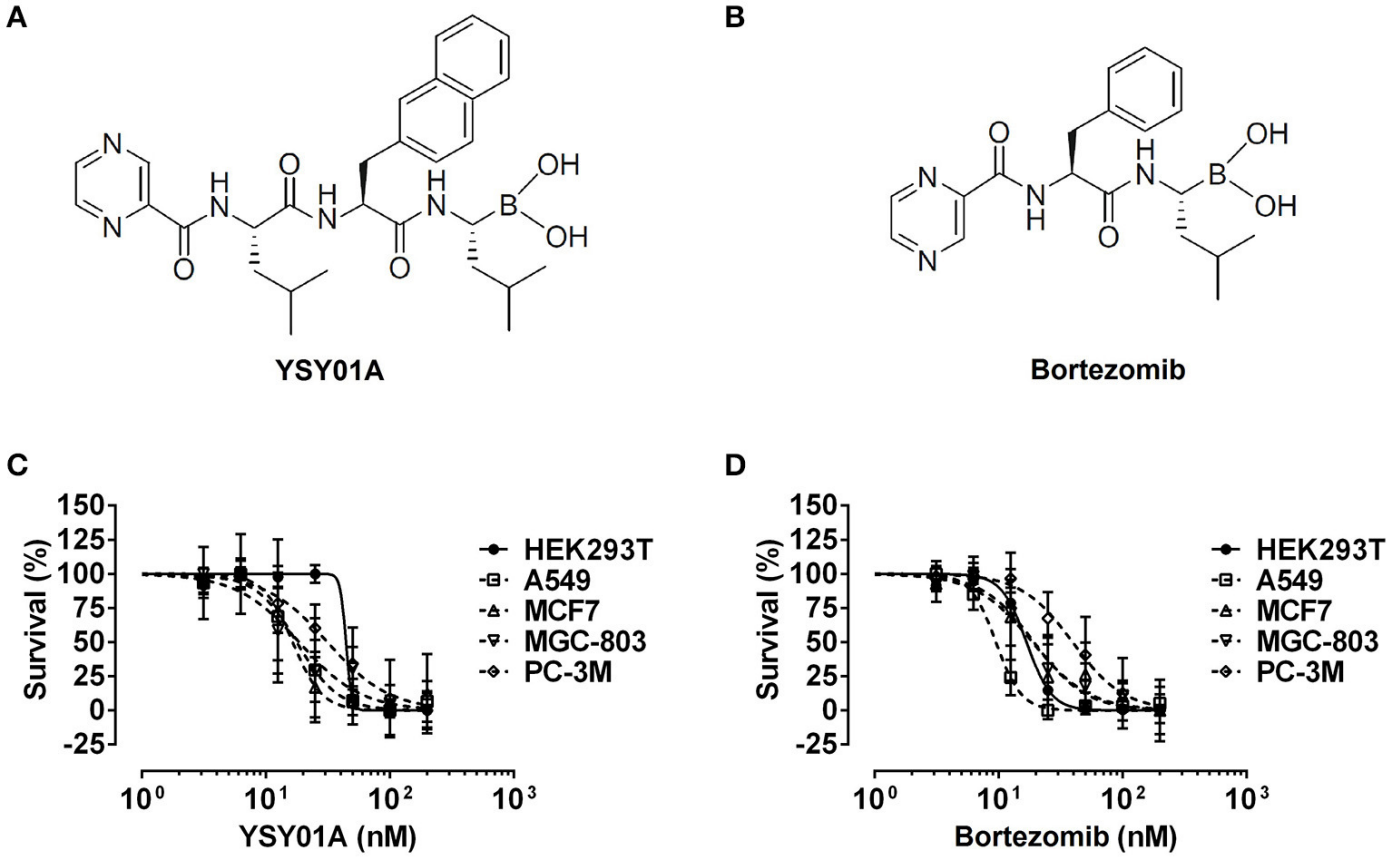
Cytotoxicity of YSY01A and Bortezomib in cell line panel. **(A)** Chemical structure of YSY01A. *N*-(2-pyrazinecarbonyl)-*L*-leucine-*L*-(2-naphthyl)-alanine-*L*-leucineboronic acid, formula:C_29_H_38_BN_5_O_5_, molecular mass: 547.45 g/mol. **(B)** Chemical structure of bortezomib (PS-341). [(1*R*)-3-methyl-1-({(2*S*)-3-phenyl-2-[(pyrazin-2-yl carbonyl)amino]propanoyl}amino)butyl] boronic acid, formula: C_19_H_25_BN_4_O_4_, molecular mass: 387.237 g/mol. **(C**,**D)** HEK293T, A549, MCF-7, MGC-803 and PC-3M cells were subjected to SRB assay after exposure to increasing concentrations of YSY01A **(C)** or bortezomib **(D)** for 72 h. YSY01A showed an *in-vitro* therapeutic window of 1.4~3.4 fold rather than bortezomib.

YSY01A and bortezomib were synthesized as we previously described (Figures 1A,B; Zhu et al., 2009). Both compounds were dissolved in dimethyl sulfoxide (DMSO) to a concentration of 10 mM and preserved at –20°C. 3-methyladenine (3-MA) was purchased from Selleck Chemicals (Houston, TX, USA). Bafilomycin A1 (Baf A1) was obtained from KeyGEN Biotech (KeyGEN, Nanjing, China). Antibodies against STAT3 (Cat. No. 9139), pSTAT3 (Cat. No. 9145), JAK2 (Cat. No. 3230), cyclin D1 (Cat. No. 2798), survivin (Cat. No. 2808), caspase-3 (Cat. No. 9662), cleaved poly (ADP-ribose) polymerase (c-PARP, Cat. No. 5625), LC3 (Cat. No. 3868), beclin-1 (Cat. No. 3738), and β-actin (Cat. No. 4970) were purchased from Cell Signaling Technology (Danvers, MA, USA). Antibodies against glycoprotein 130 (gp130; Cat. No. sc-656) and GAPDH (Cat. No. sc-25778) were obtained from Santa Cruz Biotechnologies (Santa Cruz, CA, USA).

### Cell culture and cell viability assay

Human non-small cell lung cancer cell line A549, breast cancer cell line MCF7, gastric cancer cell line MGC-803 and prostate cancer cell line PC-3M were cultured in RPMI 1640 medium (Macgene, Beijing, China), supplemented with 10% fetal bovine serum (FBS; PAN-Biotech GmbH, Aidenbach, Germany) and 1% penicillin/streptomycin (Macgene) in a 37°C humidified atmosphere containing 5% CO_2_. Human embryonic kidney cells HEK293T were maintained in DMEM containing 10% FBS and 1% penicillin/streptomycin (Macgene) in a 5% CO_2_ incubator at 37°C. Sulforhodamine B (SRB) assay was used to determine the cytotoxicity of YSY01A and bortezomib after each cell line was exposed to YSY01A or bortezomib for 72 h as we previously described (Huang et al., 2014, 2016a). Dose-response curves and IC_50_ values were computed by using GraphPad software (La Jolla, CA, USA).

### Flow cytometric analysis of apoptosis

For quantification of YSY01A-induced apoptosis, cells were labeled with Annexin V and propidium iodide (PI) by using Annexin V/PI Apoptosis Detection Kit (DOJINDO LABORATORISE, Shanghai, China) according to the manufacturer's instructions as previously described (Huang et al., 2016b). Cells stained with Annexin V and PI were then analyzed by BD Accuri^TM^ C6 Flow Cytometer (BD Biosciences, San Jose, CA, USA) to detect apoptosis in cells.

### Cell death enzyme-linked immunosorbent assay (ELISA)

Apoptosis was also verified by using Cell Death Detection ELISA^PLUS^ Photometric Enzyme Immunoassay Kit (Roche Diagnostics GmbH, Mannheim, Germany). In brief, 1 × 10^4^ cells were harvested and lyzed in 200 μl of lysis buffer provided by the kit for 30 min at room temperature followed by centrifugation at 200 × g for 10 min. 20 μl of supernatant was then added to ELISA microplate for quantification of cytoplasmic histone-associated DNA fragments according to the manufacturer's instructions.

### Real-time cell analysis (RTCA)

The effect of YSY01A on cell migration was assessed by using xCELLigence RTCA DP (ACEA Biosciences, San Diego, CA USA), which was placed in a humidified incubator at 37°C with 5% CO_2_. 4 × 10^4^ cells per well were seeded into the upper compartments of a two-chamber instrument (CIM-plate 16, ACEA Biosciences) with serum-free medium in the absence or presence of YSY01A. Medium containing 10% FBS in the lower chambers serves as a chemo-attractant. Two chambers were separated by a porous membrane, which allows cells to pass through the 8-μm pores on the chamber membrane. During 12-h incubation at 37°C, the migrating cells attached on the undersurface of the membrane were monitored by measuring the electrical impedance of integrated gold microelectrodes. The electrical impedance was eventually displayed as a dimensionless parameter termed cell index. The cell index which represents the capacity of cell migration was automatically determined every 10 min for up to 12 h using RTCA software (ACEA Biosciences).

### Western blotting

To determine the protein expression level, we performed Western blotting analysis as previously described (Huang et al., 2014, 2016a). Briefly, cells were harvested, washed and lysed using 50 mM Tris·HCl, pH 7.4, 150 mM NaCl, 20 mM EDTA, 0.5% NP-40, 1 mM Na_3_VO_4_, 50 mM NaF, 1 mM DTT and 2 mM PMSF. After 10-min incubation on ice and brief sonication, protein lysates were subjected to centrifugation at 14,000 × g using Heraeus Multifuge X1R centrifuge (Thermo Scientific, Osterode, Germany) at 4°C for 20 min. The concentration of the supernatant was measured by Bicinchoninic Acid Protein Assay Kit (Dingguo Changsheng Biotechnology, Beijing, China). Equal amount of protein lysates were separated by sodium dodecyl sulfate-polyacrylamide gel electrophoresis and electroblotted onto polyvinylidene difluoride membranes (EMD Millipore, Billerica, MA, USA). The membrane was then probed with antibodies against different proteins and visualized by using ChemiDoc XRS^+^ imaging system (Bio-Rad, Hercules, CA, USA) with enhanced chemiluminescence (Dingguo Changsheng Biotechnology). The relative protein levels were determined by the density of Western blot bands as measured by Image J software and normalized against the internal control GAPDH or β-actin.

### Real-time reverse transcription polymerase chain reaction (PCR)

Real-time qPCR was performed to determine the mRNA expression levels as we previously described (Huang et al., 2014, 2016a). Total RNA was isolated from cells using E.Z.N.A.^TM^ Total RNA Kit I (Omega Bio-tek, Norcross, GA, USA) followed by reverse transcription to cDNA using All-In-One RT MasterMix Kit (Applied Biological Materials, Richmond, BC, Canada). The resultant cDNA was subjected to 40 cycles of PCR amplification on Stratagene Mx3000 system (Agilent, Santa Clara, CA, USA) using EvaGreen qPCR MaxterMix (Applied Biological Materials) under the following cycling condition: 95°C for 10 min and 40 cycles of 95°C for 15 s and 60°C for 1 min. The primers for the genes of interest were shown as follows: bcl-2: forward, 5′-ATCGCCCTGTGGATGACTGAG-3′, reverse, 5′-CAGCCAGGAGAAATCAAACAGAGG-3′; cyclin D1: forward, 5′-CTTCCTCTCCAAAATGCCAG-3′, reverse, 5′-AGAGATGGAAGGGGGAAAGA-3′; GAPDH: forward, 5′-AAGGACTCATGACCACAGTCCAT-3′, reverse, 5′-CCATCACGCCACAGTTTCC-3′; gp130: forward, 5′-TCTGGGAGTGCTGTTCTGCTT-3′, reverse, 5′-TGTGCCTTGGAGGAGTGTGA-3′; JAK2: forward, 5′-TTTGGCAACAGACAAATGGA-3′, reverse, 5′-GCAGGAAGCTGATGCCTATC-3′; STAT3, forward, 5′-GGCCCCTCGTCATCAAGA-3′, reverse, 5′-TTTGACCAGCAACCTGACTTTAGT-3′; survivin, forward: 5′-TGCCTGGCAGCCCTTTC-3′, reverse, 5′-CCTCCAAGAAGGGCCAGTTC-3′. GAPDH was used as the internal control. The relative mRNA levels were determined using 2^−ΔΔCt^ method.

### *In-vivo* efficacy analysis using xenograft mouse model

This animal study was carried out in accordance with the recommendations of “Regulations for the administration of affairs concerning experimental animals” and related laws and regulations, and approved by Department of Laboratory Animal Science, Peking University Health Science Center. 1 × 10^7^ A549 cells were injected subcutaneously in the flanks of 21 male BLAB/c nu/nu nude mice aged 5~6 weeks. When the tumor volumes reached about 50.0 mm^3^, these mice were randomly divided into three groups and received intraperitoneal injection of vehicle control, YSY01A (0.5 mg/kg) or cisplatin (1.5 mg/kg) every 4 days. Tumor volume and body weight of the mice in each group were measured on the day of dosing without blinding. After the treatment, mice were euthanized and tumor tissues were dissected and weighed. Necropsy was also performed to determine changes in the liver and spleen.

### Immunohistochemistry staining

Immunohistochemistry analysis was used to verify the molecular alternation in paraffin-embedded tissue sections as we previously described (Huang et al., 2014). In brief, 4-μm thick tissues were dewaxed in xylene overnight and rehydrated in a gradient concentration of ethanol, followed by immersing in 3% H_2_O_2_ at room temperature for 5 min to block endogenous peroxidase activity and target retrieval buffer (10 mM citrate acid, 0.05% Tween-20, pH 6.0) at 95–100°C for 30 min. Following incubation with phosphate-buffered saline containing 10% blocking serum (Solarbio Life Sciences, Beijing, China) and 0.1% Tween-20 for 30 min at room temperature, the tissue sections were incubated at 4°C overnight with anti-STAT3 or anti- survivin antibody followed by further incubation with secondary antibody and DAB-peroxidase substrate (GeneTech, Shanghai, China). Slides were finally counterstained with hematoxylin and mounted with coverslips, and images were captured under a microscope.

### Statistical analyses

Data were presented as mean ± standard deviation. The significant difference between two groups were analyzed by the Student's *t*-test. Statistical significance was considered at *p* < 0.05.

## Results

### YSY01A inhibits cancer cell survival by inducing apoptosis

The novel proteasome inhibitor, YSY01A, has shown a broad spectrum of antitumor activity (Wang et al., 2014; Xue et al., 2015; Zhang et al., 2015; Huang et al., 2016b). In this study, we further assessed the cytotoxicity of YSY01A in four cancer cell lines including A549 (lung cancer), MCF-7 (breast cancer), MGC-803 (gastric cancer) and PC-3M (prostate cancer) cells and non-cancerous human embryonic kidney cells HEK293T by using SRB assay following YSY01A treatment for 72 h. As showed in Table 1 and Figure 1C, IC_50_ values of YSY01A ranged from 15.2 to 35.4 nM for cancer cells and the cancer cells appeared to be more sensitive to YSY0A1 as compared with non-cancerous HEK293T cells. This finding of difference in IC_50_ values suggests a potential *in-vitro* therapeutic window for YSY01A. In contrast, bortezomib, the FDA-approved proteasome inhibitor, showed little difference in IC_50_ between HEK293T and cancer cells (Figure 1D). Despite the need of more evidences on normal cells, YSY01A warrants further investigations to verify the potential advantage of YSY01A in the context of cancer.

**Table 1:** IC_50_ of YSY01A and Bortezomib.

**Cell line**	**Histology**	**IC**_**50**_ **(nM)**	
		**YSY01A**	**Bortezomib**
HEK293T	Human embryonic kidney cells	51.0 ± 11.5	16.5 ± 2.0
A549	Human lung adenocarcinoma	19.2 ± 9.8^*^	9.5 ± 1.4^*^
MCF-7	Human breast cancer	15.2 ± 11.9^*^	20.1 ± 7.1
MGC-803	Human gastric cancer	18.9 ± 7.4^*^	20.9 ± 7.7
PC-3M	Human prostate cancer	35.4 ± 16.5	42.1 ± 19.5

*Data are represented as mean ± standard deviation*.

* *p < 0.05, by Student's t-test as compared with corresponding IC_50_ value of HEK293T cells*.

**Figure 2:**
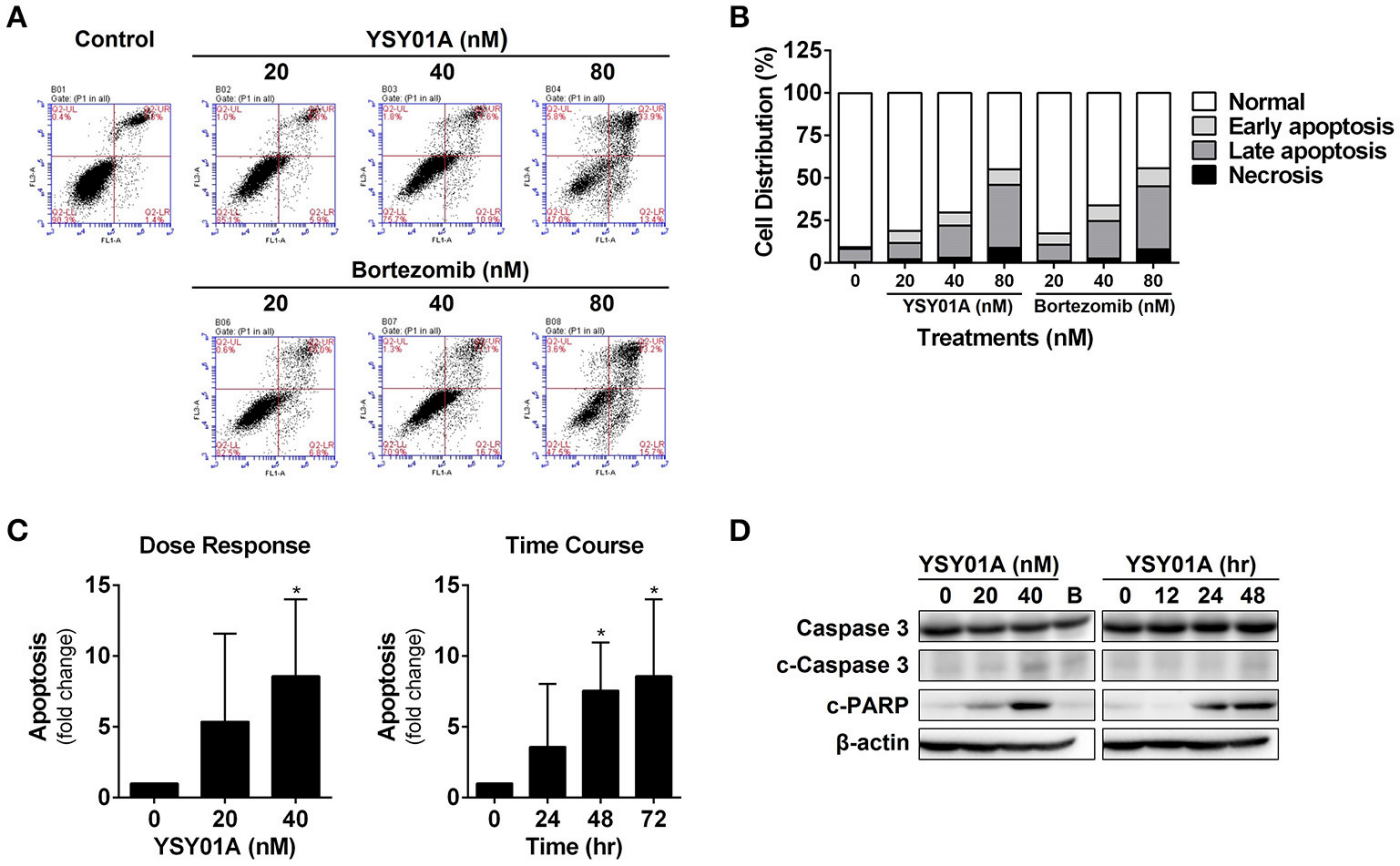
YSY01A induces apoptosis in A549 cells. **(A)** Following treatment with 0 (vehicle control), 20, 40 nM or 80 nM YSY01A or bortezomib for 48 h, A549 cells were subjected to Annexin V/PI staining and flow cytometry analysis to determine apoptotic cells. Shown is a representative of flow cytometry data. Viable cells (left bottom quadrant, Annexin V^−^/PI^−^), early apoptotic cells (right bottom quadrant, Annexin V^+^/PI^−^), late apoptotic cells (right upper quadrant, Annexin V^+^/PI^+^) and necrotic cells (left upper quadrant, Annexin V^−^/PI^+^). **(B)** Percentages of cells in each quadrant are shown. **(C)** YSY01A-induced apoptosis was determined using the Cell Death Detection ELISA^PLUS^ Kit. (^*^p < 0.05, by Student's *t*-test as compared with vehicle control) **(D)** Immunoblotting with cleavage of caspase 3 and PARP. β-actin was used as a loading control. B, bortezomib.

To determine if YSY01A suppression of cancer cell survival results from apoptosis, we performed flow cytometric assay to quantify apoptotic cells following treatment with YSY01A. As evident from Figures 2A,B, early and late apoptotic cells accounted for 12.3, 20.1, and 44.7% upon treatment of A549 cells with 20, 40, and 80 nM YSY01A for 48 h, respectively. The percentages of apoptotic cells increased to 15.8, 24.1, and 46.5% following treatment with 20, 40, and 80 nM bortezomib, respectively, as compared to untreated cells. ELISA analysis, which quantifies cytoplasmic histone-associated DNA fragments released from apoptotic cells, also supported induction of apoptosis in A549 cells following YSY01A treatment in dose- and time-dependent manners (Figure 2C). Moreover, YSY01A treatment also induced cleavage of caspase 3 and PARP in A549 cells, two key executioners of apoptosis, confirming YSY01A-induced apoptosis (Figure 2D and Figure S1).

### YSY01A abrogates constitutive activation of STAT3

**Figure 3:**
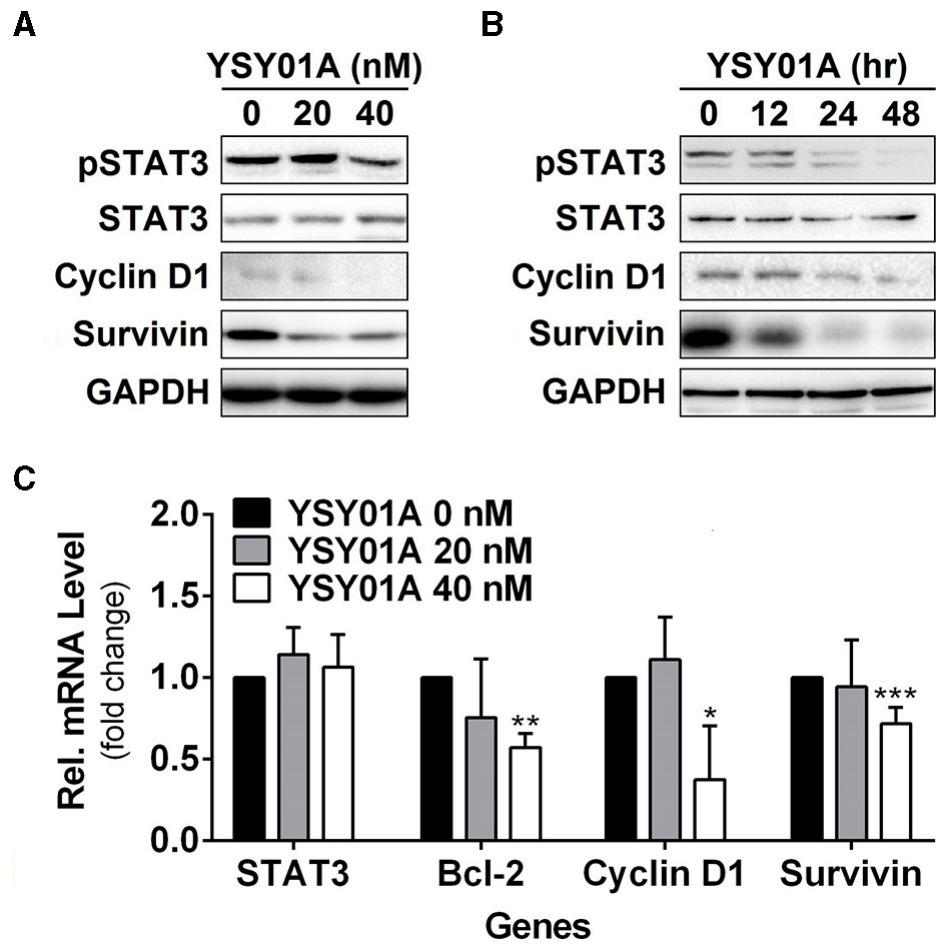
Effect of YSY01A on regulatory proteins important for cell proliferation and anti-apoptosis in A549 cells. **(A)** Expression levels of specific proteins important for cell proliferation and anti-apoptosis in a dose-response study. 24 h after seeding, A549 cells were exposed to 0 (vehicle control), 20 or 40 nM YSY01A for 48 h followed by sample collection and immunoblot analysis. GAPDH was used as a loading control. **(B)** Expression levels of specific proteins important for cell proliferation and anti-apoptosis in a time-course experiment. 24, 48 or 60 h after seeding, A549 cells were exposed to 40 nM YSY01A for 48, 24 or 12 h, respectively. Vehicle-treated cells were used as the 0-h control. At the end of study, all cell samples were harvested and subjected to immunoblot analysis of specific protein expression. GAPDH was used as a loading control. **(C)** Following the treatment as indicated, A549 cells were harvested for real-time qPCR analysis of selected genes. GAPDH was used as an internal control. (*p < 0.05, **p < 0.01, ***p < 0.001, by Student's *t*-test as compared with vehicle control).

Constitutively activated STAT3 promotes cell proliferation and survival in many cancers including breast, brain, colon, prostate, lung, pancreatic, gastric cancers, and so on (Bowman et al., 2000). To investigate the potential effect of YSY01A on constitutively active STAT3 signaling cascade, A549 cells were cultured in the presence of YSY01A (20 and 40 nM) for 48 h or 40 nM YSY01A for different time intervals. As evident from Figures 3A,B and Figure S2, YSY01A treatment inhibits constitutive phosphorylation of STAT3 at Y705 and the expression of STAT3 downstream targets in dose- and time-dependent manners, without substantial alternation of total STAT3 protein expression. Meanwhile, the mRNA level of STAT3 remains unchanged as determined by real-time qPCR analysis, suggesting a protein-level regulation on these proteins (Figure 3C). To validate the STAT3-inhibitory activity of YSY01A, we also examined the expression of STAT3 downstream genes by using real-time qPCR. As expected, the mRNA levels of known STAT3 downstream genes including bcl-2, cyclin D1 and survivin were all decreased following YSY01A treatment in A549 cells (Figure 3C). Considering that these genes are well known to favor cancer cell proliferation and anti-apoptosis, our data suggest that YSY01A may exhibit anti-tumor effect via modulating STAT3 signaling, thereby leading to transcriptional down-regulation of STAT3 downstream genes.

### YSY01A downregulates Gp130 and JAK2 in A549 cells

**Figure 4:**
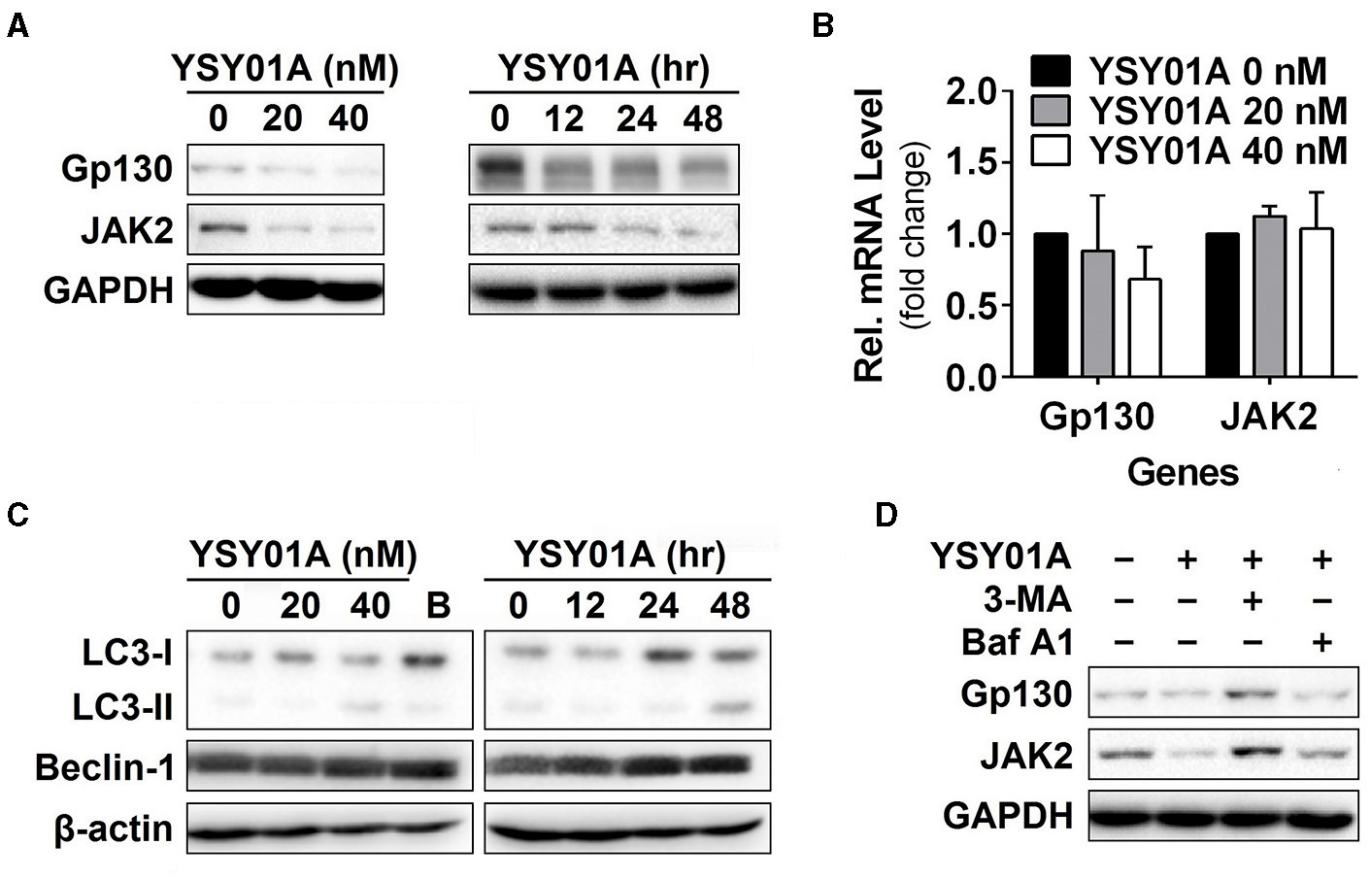
Gp130 and JAK2 are degraded by the autophagy lysosome pathway. **(A)** For the dose-response study, 24 h after seeding, A549 cells were exposed to 0 (vehicle control), 20 or 40 nM YSY01A for 48 h followed by sample collection and immunoblot analysis. GAPDH was used as a loading control. For the time-course experiment, 24, 48 or 60 h after seeding, A549 cells were exposed to 40 nM YSY01A for 48, 24 or 12 h, respectively. Vehicle-treated cells were used as the 0-h control. At the end of study, all cell samples were harvested and subjected to immunoblot analysis of specific protein expression. GAPDH was used as a loading control. **(B)** cDNA from A549 cells treated with 0 (vehicle control), 20 or 40 nM YSY01A for 48 h was evaluated for mRNA expression of gp130 and JAK2. Data are relative to GAPDH and normalized to cells treated with vehicle control. **(C)** Effects of YSY01A and bortezomib on LC3 and beclin 1, two markers of autophagy. B: bortezomib. **(D)** In the presence of proteasome inhibition, A549 cells were co-treated with 5 mM 3-methyladenine (3-MA) or 100 nM Baflomycin A1 (Baf A1) for 48 h. Whole cell lysates were evaluated for gp130, JAK2, and GAPDH by immunoblot analysis.

Given that YSY01A inhibits activation of STAT3 signaling, we hypothesized that YSY01A may affect its upstream components and similarly examined the effect of YSY01A on gp130 and JAK2 expression. For this purpose, A549 cells were exposed to increasing concentrations of YSY01A for 48 h or 40 nM YSY01A for different time intervals followed by Western blotting and qPCR analyses. As shown in Figures 4A,B and Figure S3, two STAT3 upstream components, gp130 and JAK2, were decreased at the protein level in dose- and time- dependent manners whereas their mRNA levels were not changed substantially. The present study found conversion of LC3-I to LC3-II and elevation of beclin 1 upon YSY01A treatment, which is consistent with the findings of bortezomib (Figure 4C). Under stressed conditions, lysosomal-mediated degradation of selected cellular components has been shown to contribute to cytotoxic effects of bortezomib (Fang et al., 2012). To determine whether YSY0A1-induced degradation of gp130 and JAK2 occurs as a consequence of autophagy, 3-MA, an inhibitor of autophagy, was used to evaluate whether gp130 and JAK2 degradation is reversible in the presence of YSY01A. As an early-stage autophagic lysosme and type I/III PI3K inhibitor, 3-MA restored gp130 and JAK2 protein expression in A549 cells (Figure 4D). Surprisingly, Baf A1, a late-stage autophagy inhibitor that blocks vacuolar-type H^+^-ATPase, did not prevent the degradation of gp130 and JAK2 (Figure 4D). Given the dramatic effect of 3-MA on gp130 and JAK2, we propose a posttranscriptional mechanism that regulates gp130 and JAK2 protein levels by YSY01A.

### YSY01A inhibits cell migration in A549 cells

**Figure 5:**
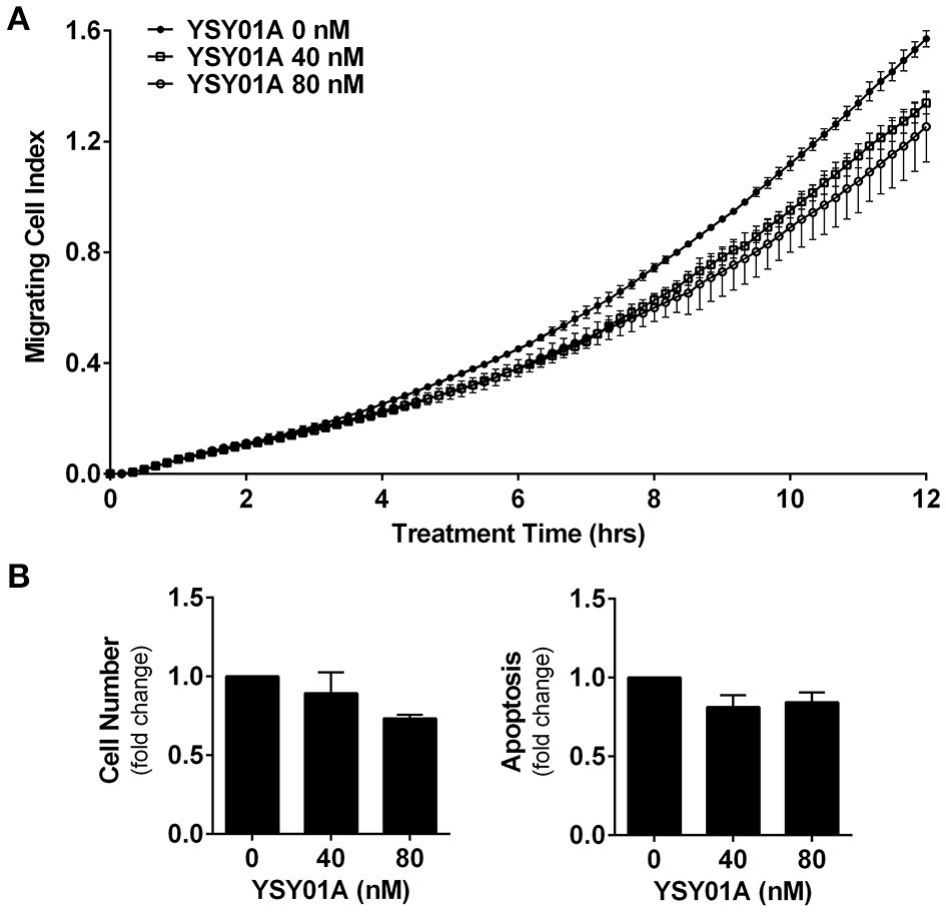
YSY01A inhibits cell migration. **(A)** Cell migration potential of A549 cells was assessed in the absence or presence of 40 or 80 nM YSY01A by using transwell migration assay after 12-h treatment. Real-time measurement of cell migration was performed using the xCELLigence DP system. Shown is a representative of cell migration data. **(B)** 100% confluent A549 cells were treated with 0, 40, or 80 nM YSY01A for 12 h followed by determination of change in cell number for cell survival (left) or by apoptosis ELISA assay (right).

Numerous studies have shown that STAT3 signaling cascade plays an important role in controlling cell migration by regulating the expression of its downstream genes such as twist and matrix metalloproteinases important for this cellular process (Devarajan and Huang, 2009). To investigate the effect of YSY01A on migratory ability of A549 cells, we performed a transwell migration assay using A549 cells. As the conventional transwell migration assay is time-consuming with inevitable bias, the migrating process was monitored by using real-time cell analyzer which enables real-time and automatic measurement (Roshan Moniri et al., 2015). Figure 5A shows that YSY01A inhibits migration of A549 cells in dose- and time-dependent manners. Treatment with 40 and 80 nM YSY01A for 6 h reduced migration to 86 and 71%, respectively. Cell migration remained 83 and 72% after exposure to 40 and 80 nM YSY01A for 12 h, respectively (*p* < 0.01). To rule out the possibility that inhibition of proliferation and induction of apoptosis contributes to reduced migration, we used 100% confluent cells and observed the inhibition of migration within 12 h of treatment. Additionally, cell proliferation and apoptosis under the same condition as transwell migration assay were determined as well. Figure 5B shows that treatment with 40 and 80 nM YSY01A for 12 h has little effect on proliferation and apoptosis of 100% confluent A549 cells. Thus, YSY01A inhibition of cancer cell migration may not be due to its effect on cell proliferation and apoptosis.

### YSY01A inhibits tumor growth *in vivo*

**Figure 6:**
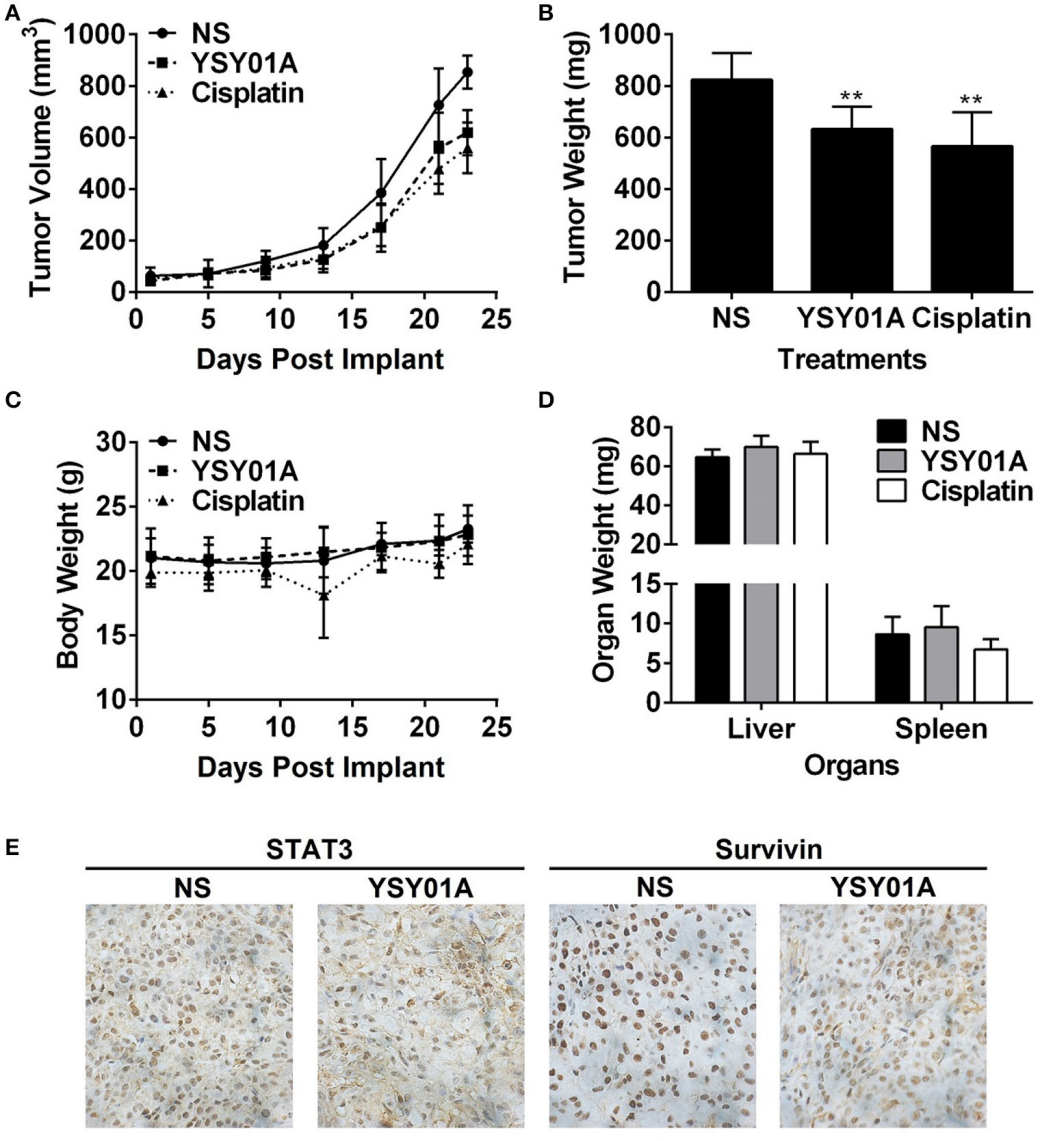
YSY01A suppresses xenograft tumor growth *in vivo*. **(A)** Volume of xenograft tumors and body weight of mice following implantation and treatments with normal saline (NS), 0.5 mg/kg YSY01A or 1.5 mg/kg cisplatin (positive control) by intraperitoneal injection. **(B)** Wet weight of finally dissected xenograft tumor mass. (^**^p < 0.01, by Student's *t*-test as compared with vehicle control) **(C)** Body weight of mice after implantation and treatments. **(D)** Wet weight of finally dissected organs. **(E)** Immunohistochemistry staining of xenograft tumor tissues for the expression of STAT3 and survivin.

The *in-vivo* efficacy of YSY01A was evaluated using a mouse xenograft model of A549 cells. As shown in Figures 6A,B, intraperitoneal injection of YSY01A significantly reduced tumor growth as compared with the vehicle control treatment. Moreover, no significant change in body weight existed during the course of treatment (Figure 6C). YSY01A-treated mice showed lower final tumor weight than normal saline-treated group with little effect on the wet weight of liver and spleen (Figure 6D). Immunohistochemistry staining analysis of xenograft tumors also revealed decreased staining of STAT3 downstream target gene, survivin, in YSY01-treated tumors, but not total STAT3 itself (Figure 6E). These findings suggest that YSY01A inhibits xenograft tumor growth with little adverse effect.

## Discussion

YSY01A has been reported to have antitumor effects *in vitro* and *in vivo* by modulating various signaling pathways involving in cell proliferation, cell cycle regulation, autophagy and apoptosis (Wang et al., 2014; Xue et al., 2015; Zhang et al., 2015; Huang et al., 2016b). YSY01A also enhances cisplatin cytotoxicity in cisplatin-resistant ovarian cancer by suppression of NF-κB and STAT3 targeted genes such as bcl-2 (Huang et al., 2016b). However, comparatively few studies have explored the therapeutic efficacy of YSY01A in non-small cell lung carcinoma and little is known about the mechanism by which YSY01A modulates STAT3 signaling cascade. In the present study, we reported that YSY01A suppresses survival of tumor cells including A549 (lung cancer), MCF-7 (breast cancer), MGC-803 (gastric cancer) and PC-3M (prostate cancer) cells. Although YSY01A showed comparable *in-vitro* cytotoxicity on the tested cancer cell lines, the compound is less toxic to non-cancerous HEK293T cells as comparable as bortezomib, the FDA-approved drug. The presence of a potential therapeutic window warrants further studies whereas more studies on normal cells and *in-vivo* toxicity are also needed. More, YSY01A exhibited an *in-vivo* efficacy in a mouse xenograft model of A549 cells with little effect on liver and spleen. An ongoing extensive study on *in-vivo* efficacy of YSY01A will bring more insights to clinical value of this compound. We further investigated the molecular and cellular consequences and found that YSY01A abrogates constitutive activation of STAT3 by degradation of gp130 and JAK2 via autophagic lysosomes, leading to down-regulation of STAT3 downstream genes. The decline in survivin expression, one of STAT3 downstream targets, was verified *in vivo* as well. In addition, we show that YSY01A treatment contributes to the inhibitory effect on cell migration. These findings show promise in further modification and development of YSY01A as a novel proteasome inhibitor.

STAT3 is a transcription factor, which tightly regulates signaling processes through extracellular cytokines and growth factors (Zhong et al., 1994). Constitutive activation of STAT3 has been demonstrated to be sufficient for inducing oncogenesis (Bromberg et al., 1999). STAT3 is frequently activated in a variety of cancers and plays important roles in multiple aspects of cancer aggressiveness. The STAT3 signaling pathway is especially important for cell proliferation, anti-apoptosis, chemoresistance, metastasis and angioneisis, and cancer immune evasion through constitutive phosphorylation of STAT3 or in response to interleukin-6 (IL-6) provided by cells in the bone marrow microenvironment or by tumor cells (Zhong et al., 1994). Bortezomib has been shown to induce caspase-dependent down-regulation of gp130, thereby abrogating IL-6-triggered signaling cascades (Hideshima et al., 2003). Bortezomib triggers caspase activation and induces cleavage of the cytoplasmic tail of gp130 located within position 800–806 (DHVDGGD), leading to inhibition of IL-6 induced STAT3 phosphorylation (Graf et al., 2008). Although, we also found YSY01A inhibits constitutive activation of STAT3 and down-regulation of STAT3 upstream components, gp130 and JAK2, cotreatment with YSY01A and pan-caspase inhibitor Z-VAD-FMK failed to restore down-regulation of gp130 and JAK2 (Figure S3F). Apparently, YSY01A may elicit a different mechanism resulting in protein down-regulation in A549 cells. One possibility is autophagic lysosome pathway. Bortezomib and related proteasome inhibitors have been shown to activate autophagy in various cancers (Lou et al., 2013; Selimovic et al., 2013). With persistent bortezomib-induced stress, the proteins are sequestered to lysosomes, leading to the degradation. For example, Fang et al. found that cytotoxic effects of bortezomib depend on autophagy-medicated lysosomal degradation of TRAF6 (Fang et al., 2012). YSY01A has been shown to induce autophagy in prostate cancer cells (Wang et al., 2014). Coincidentally, the present study showed that 3-MA protected YSY01A-treated cells from down-regulation of gp130 and JAK2 but not Baf A1. YSY01A-induced conversion of LC3 to the lower migrating form, LC3-II, suggesting that YSY01A may activate signaling cascades that initiate autophagosome generation. In fact, lysosomal degradation of key regulation in cells may be involved in cytotoxic effects of proteasome inhibitors, and the use of autophagy modulators in cancer therapy needs to be evaluated carefully. Additionally, there are ongoing efforts to uncover the molecular mechanisms of YSY01A as down-regulation of gp130 and JAK2 may be likely due to the shut-down of *de novo* protein synthesis as well. Proteasome inhibition results in the accumulation of unfolded or misfolded proteins in endoplasmic reticulum (ER), leading to ER stress (Zhang et al., 2016). In response to the ER stress, the phosphorylation of the ER membrane-resident kinase PERK was activated, leading to eIF2α phosphorylation and eventually a general inhibition of protein synthesis. It has been reported that bortezomib-induced ER stress directly relates to the shut-down of *de novo* synthesis (Obeng et al., 2006). Nevertheless, more studies are needed to reveal the role of YSY01A in this process.

It is noteworthy that multiple events and many protein targets are regulated by the ubiquitin-proteasome pathway. Proteasome inhibitors act on multiple targets and induce diverse cellular changes via different signaling pathways. Previous proteomic study has shown that various regulatory proteins involving in the control of cell proliferation, apoptosis, cell cycle and autophagy may be affected by YSY01A treatment (Wang et al., 2014; Xue et al., 2015; Zhang et al., 2015; Huang et al., 2016b). Unfortunately, despite the clinical success of bortezomib in multiple myeloma, an obstacle for clinical use of bortezomib is resistance to this drug as a result of gene mutation and overexpression and pharmacokinetic properties (McConkey and Zhu, 2008). Noval proteasome inhibitors might provide a solution to overcome bortezomib resistance caused by overexpression of proteasome β5 subunit (Franke et al., 2012). In previoius study, YSY01A is 5-fold more potent than bortezomib in inhibiting the trypsin-like site (β2/β2i) of the proteasome with comparable activitiy against the chymotrpsin-like site (β5/β5i) and the post-glutamyl peptide hydrolase site (β1/β1i) (Zhang et al., 2015). YSY01A undoubtedly represents an attractive candidate for development of novel proteasome inhibitors. Furthermore, YSY01A treatment significantly reduced tumor growth in A549 xenograft models. In our other studies using breast cancer and prostate cancer xenograft models, 1.25 ~ 3.25 mg/kg YSY01A also inhibited tumor growth by more than 50% without concurrent body weight loss or organ damage. Currently, an extensive study is ongoing to examine the *in-vivo* efficacy of YSY01A in a broader dose range using A549 xenograft models. Although, the tested dose in the present study only induced ~30% tumor growth inhibition, lack of apparent phenotypic changes, change in organ size and weight loss suggest a little opportunity of multiple dosing of YSY01A to cause sever adverse effects *in vivo* and thus YSY01A warrants further development. Collectively, the present study provides an extensive description of the mechanism whereby YSY01A abrogates constitutive activation of STAT3 via down-regulation of gp130 and JAK2. Collectively, the potential benefit of YSY01A in cancer therapy awaits further investigations.

We conclude that YSY01A suppresses survival of cancer cells and growth of A549 xenografts *in vivo*. The mechanisms by which YSY01A abrogates constitutive activation of STAT3 is associated with proteasome-independent degradation of gp130 and JAK2. YSY01A also induces apoptosis and inhibits cell migration. Taken together, our findings suggest that YSY01A may be developed as a promising candidate for cancer therapeutics by targeting the proteasome.

## Author contributions

WH, XY, TS, HY, RL, and JC conceived the study. WH conducted the experiments. TS performed animal efficacy study. XY, QZ, and FR assisted in cell culture. SF and JW assisted in flow cytometric analysis. WG assisted in animal efficacy study. ZG and RL were in charge of chemical synthesis. All authors discussed the results and commented on the manuscript. WH, RL, and JC analyzed the data and drafted the manuscript.

### Conflict of interest statement

The authors declare that the research was conducted in the absence of any commercial or financial relationships that could be construed as a potential conflict of interest. The reviewer OF and handling Editor declared their shared affiliation, and the handling Editor states that the process met the standards of a fair and objective review.

## Supplementary material

Figure S1 Expression levels of apoptotic factors following treatment. Quantification of Figure 2D as measured by the density of Western blot bands and normalized against the loading control. **(A)** A549 cells were treated with 0 (vehicle control), 20 or 40 nM YSY01A, or 40 nM bortizomib for 48 h. **(B)** A549 cells were treated with 40 nM YSY01A for 0, 12, 24, or 48 h.

Figure S2 Expression levels of pSTAT3 and STAT3 following treatment. Quantification of Figures 3A,B measured by the density of Western blot bands and normalized against the loading control. **(A)** A549 cells were treated with 0 (vehicle control), 20 or 40 nM YSY01A, or 40 nM bortizomib for 48 h. **(B)** A549 cells were treated with 40 nM YSY01A for 0, 12, 24, or 48 h (^*^*p* < 0.05, ^**^*p* < 0.01, ^***^*p* < 0.001, by Student's *t*-test as compared with vehicle control).

Figure S3 Expression levels of gp130 and JAK following treatment. Quantification of Figure 4 measured by the density of Western blot bands and normalized against the loading control. **(A** and **C)** A549 cells were treated with 0 (vehicle control), 20 or 40 nM YSY01A, or 40 nM bortizomib for 48 h. **(B** and **D)** A549 cells were treated with 40 nM YSY01A for 0, 12, 24, or 48 h. **(E)** In the presence of YSY01A treatment, A549 cells were co-treated with 5 mM 3-methyladenine (3-MA) or 100 nM Baflomycin A1 (Baf A1) for 48 h. **(F)** A549 cells were exposed to 40 nM YSY01A for 48 h in the absence or presence of Z-VAD-FMK, a pan-caspase inhibitor. (^*^*p* < 0.05, by Student's *t*-test as compared with vehicle control).
