# Corrigendum: Proteasome inhibitor YSY01A abrogates constitutive STAT3 signaling via down-regulation of Gp130 and JAK2 in human A549 lung cancer cells

**DOI:** 10.3389/fphar.2024.1418274

**Published:** 2024-08-05

**Authors:** Wei Huang, Xia Yuan, Ting Sun, Shujie Fan, Jun Wang, Quan Zhou, Wei Guo, Fuxiang Ran, Zemei Ge, Huayu Yang, Runtao Li, Jingrong Cui

**Affiliations:** ^1^ Department of Pharmacology, Institute of Basic Medical Sciences, Chinese Academy of Medical Sciences and School of Basic Medicine, Peking Union Medical College, Beijing, China; ^2^ State Key Laboratory of Natural and Biomimetic Drugs, School of Pharmaceutical Sciences, Peking University, Beijing, China; ^3^ Department of Medicinal Chemistry, School of Pharmaceutical Sciences, Peking University, Beijing, China; ^4^ Department of Liver Surgery, Peking Union Medical College Hospital, Chinese Academy of Medical Sciences and Peking Union Medical College, Beijing, China

**Keywords:** proteasome inhibitor, YSY01A, STAT3 signaling, protein degradation, non-small cell lung carcinoma

In the published article, there was an error in Figures 3A, B and Figures 4A, C as published. The bands of some proteins shared the same control treated with DMSO vehicle. To avoid misunderstanding, Figures 3A, B and Figures 4A, C were replaced with the images from other repetitive experiments. The corrected Figures 3, 4 and their captions appear below.

In the published article, there was an error in the legends for Figure 3 and Figure 4 as published. The captions were changed to improve clarity. The corrected legends appear below.

**FIGURE 3:**
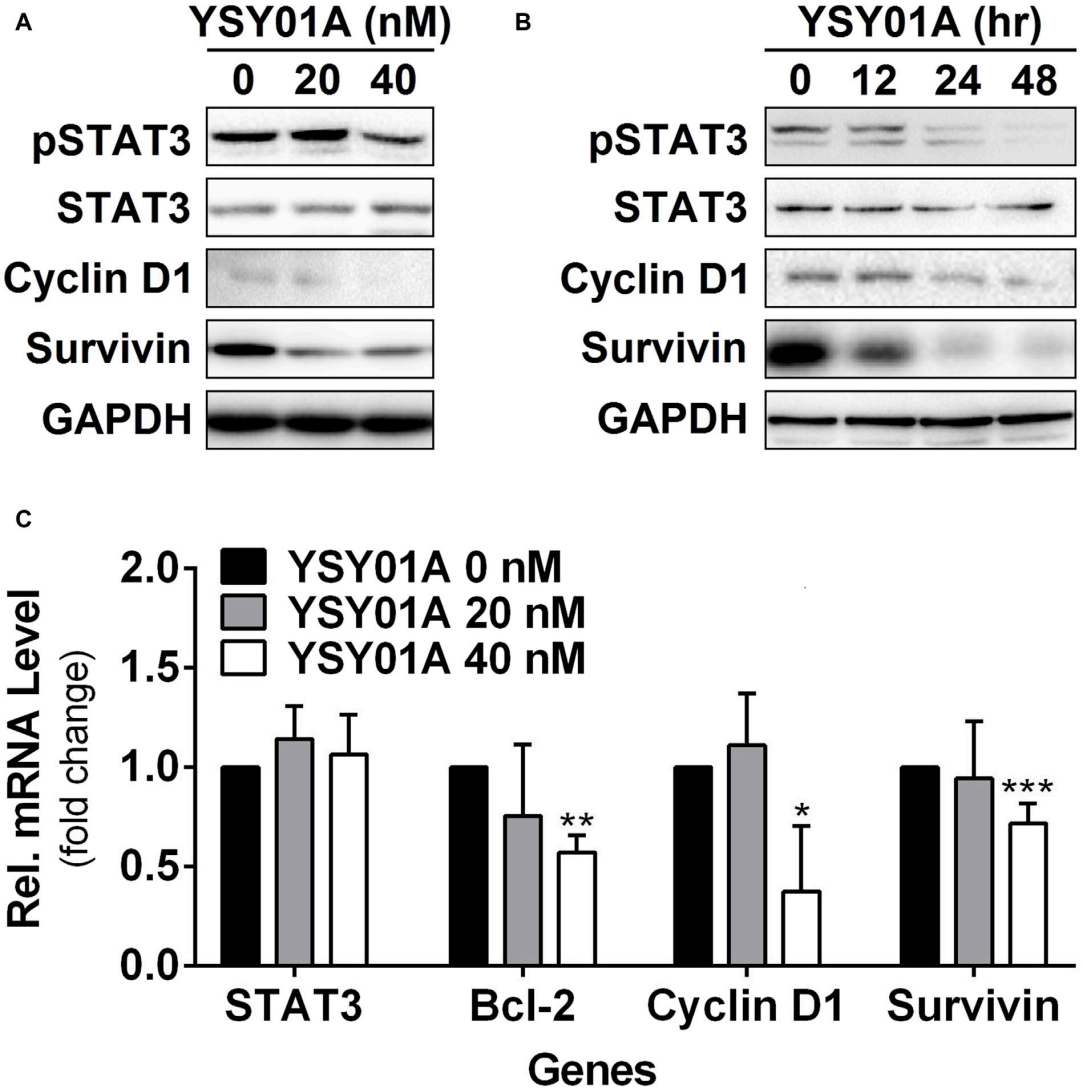
Effect of YSY01A on regulatory proteins important for cell proliferation and anti-apoptosis in A549 cells. **(A)** Expression levels of specific proteins important for cell proliferation and anti-apoptosis in a dose-response study. 24 h after seeding, A549 cells were exposed to 0 (vehicle control), 20 or 40 nM YSY01A for 48 h followed by sample collection and immunoblot analysis. GAPDH was used as a loading control. **(B)** Expression levels of specific proteins important for cell proliferation and anti-apoptosis in a time-course experiment. 24, 48 or 60 h after seeding, A549 cells were exposed to 40 nM YSY01A for 48, 24 or 12 h, respectively. Vehicle-treated cells were used as the 0-h control. At the end of study, all cell samples were harvested and subjected to immunoblot analysis of specific protein expression. GAPDH was used as a loading control. **(C)** Following the treatment as indicated, A549 cells were harvested for real-time qPCR analysis of selected genes. GAPDH was used as an internal control. (*p < 0.05, **p < 0.01, ***p < 0.001, by Student’s t-test as compared with vehicle control).

**FIGURE 4:**
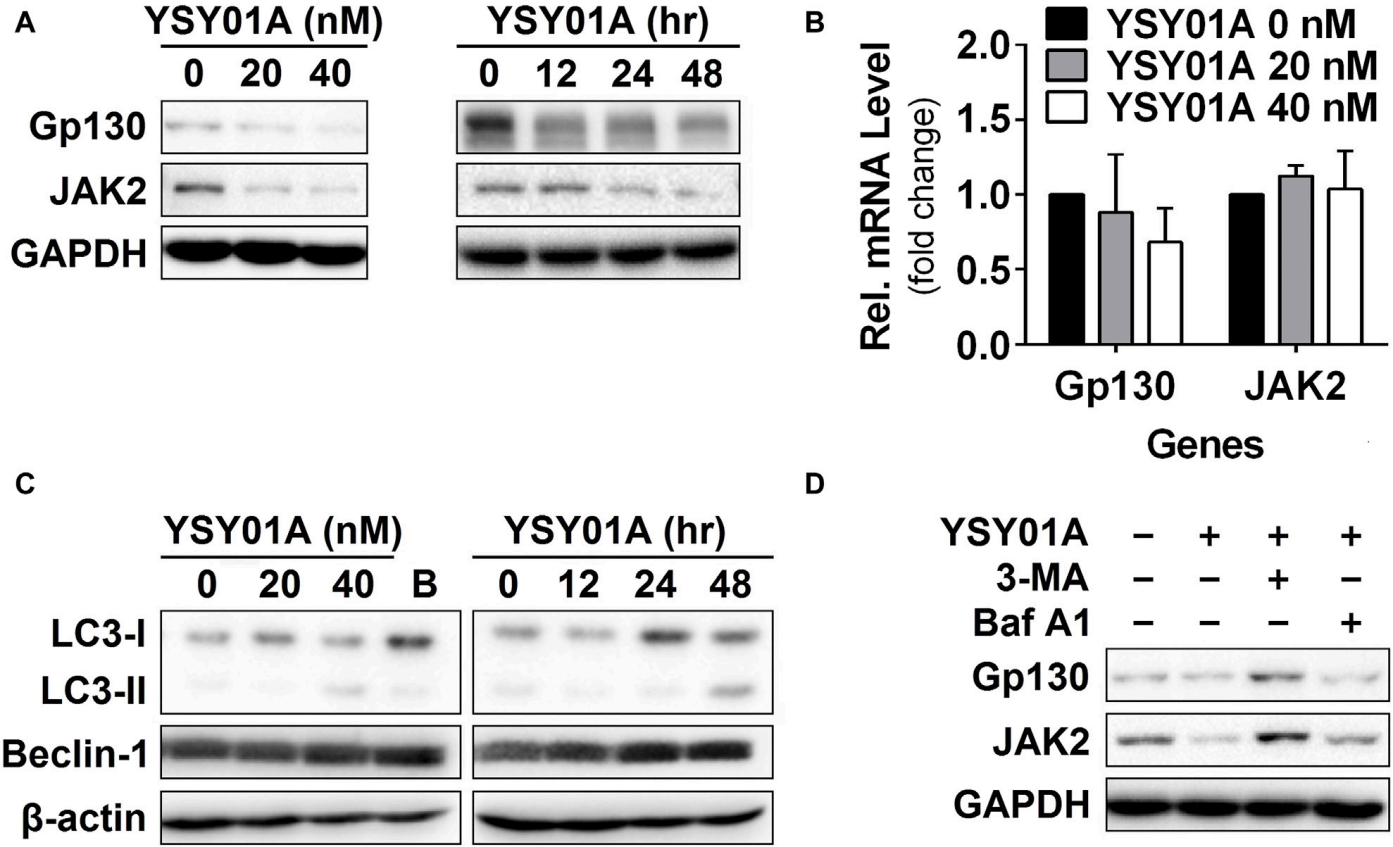
Gp130 and JAK2 are degraded by the autophagy lysosome pathway. **(A)** For the dose-response study, 24 h after seeding, A549 cells were exposed to 0 (vehicle control), 20 or 40 nM YSY01A for 48 h followed by sample collection and immunoblot analysis. GAPDH was used as a loading control. For the time-course experiment, 24, 48 or 60 h after seeding, A549 cells were exposed to 40 nM YSY01A for 48, 24 or 12 h, respectively. Vehicle-treated cells were used as the 0-h control. At the end of study, all cell samples were harvested and subjected to immunoblot analysis of specific protein expression. GAPDH was used as a loading control. **(B)** cDNA from A549 cells treated with 0 (vehicle control), 20 or 40 nM YSY01A for 48 h was evaluated for mRNA expression of gp130 and JAK2. Data are relative to GAPDH and normalized to cells treated with vehicle control. **(C)** Effects of YSY01A and bortezomib on LC3 and beclin 1, two markers of autophagy. B: bortezomib. **(D)** In the presence of proteasome inhibition, A549 cells were co-treated with 5 mM 3-methyladenine (3-MA) or 100 nM Baflomycin A1 (Baf A1) for 48 h. Whole cell lysates were evaluated for gp130, JAK2, and GAPDH by immunoblot analysis.

The authors apologize for these errors and state that this does not change the scientific conclusions of the article in any way. The original article has been updated.

