# Supplementary figures and images for: Proteasome Inhibitor YSY01A Abrogates Constitutive STAT3 Signaling via Down-regulation of Gp130 and JAK2 in Human A549 Lung Cancer Cells
Source: Front Pharmacol. 2017 Aug 24;8:476. doi: 10.3389/fphar.2017.00476 (PMC5574410; doi:10.3389/fphar.2017.00476)

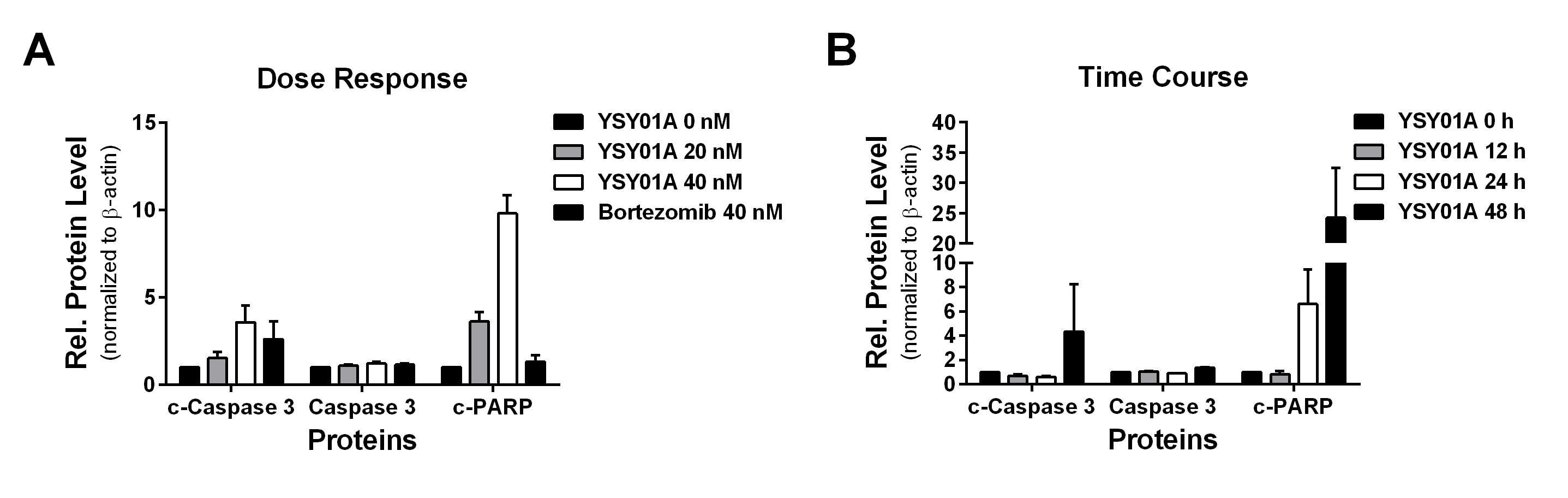

Supplement: Figure S1 — Expression levels of apoptotic factors following treatment. Quantification of Figure 2D as measured by the density of Western blot bands and normalized against the loading control. (A) A549 cells were treated with 0 (vehicle control), 20 or 40 nM YSY01A, or 40 nM bortizomib for 48 h. (B) A549 cells were treated with 40 nM YSY01A for 0, 12, 24, or 48 h. [file Image1.JPEG]

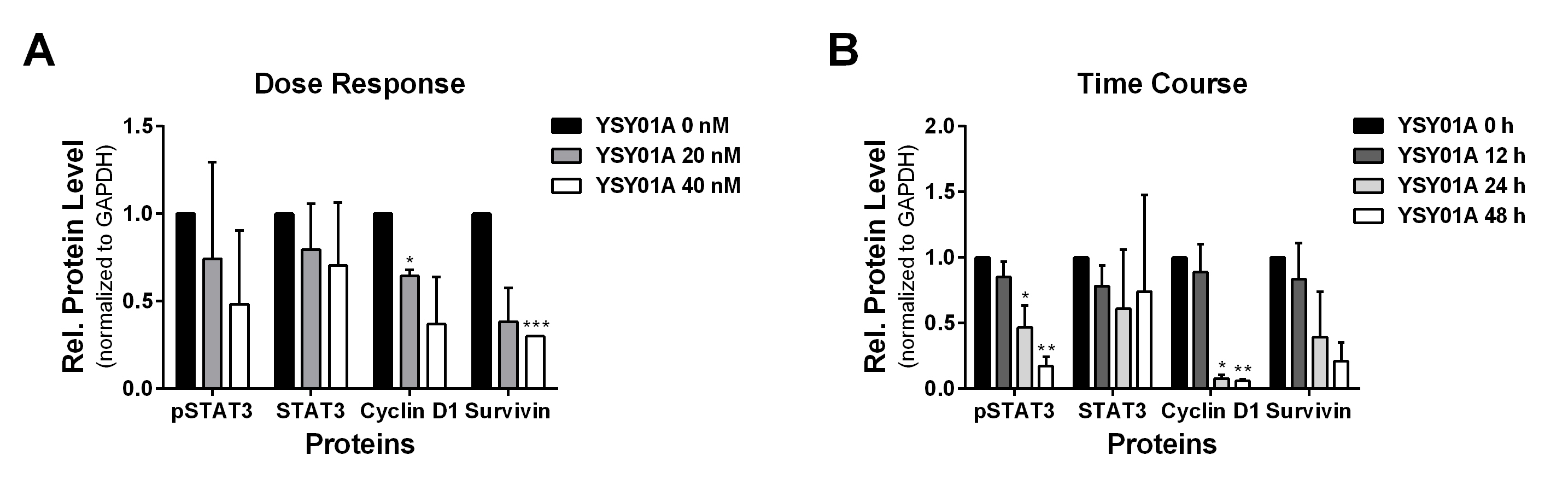

Supplement: Figure S2 — Expression levels of pSTAT3 and STAT3 following treatment. Quantification of Figures 3A,B measured by the density of Western blot bands and normalized against the loading control. (A) A549 cells were treated with 0 (vehicle control), 20 or 40 nM YSY01A, or 40 nM bortizomib for 48 h. (B) A549 cells were treated with 40 nM YSY01A for 0, 12, 24, or 48 h (*p < 0.05, **p < 0.01, ***p < 0.001, by Student's t-test as compared with vehicle control). [file Image2.JPEG]

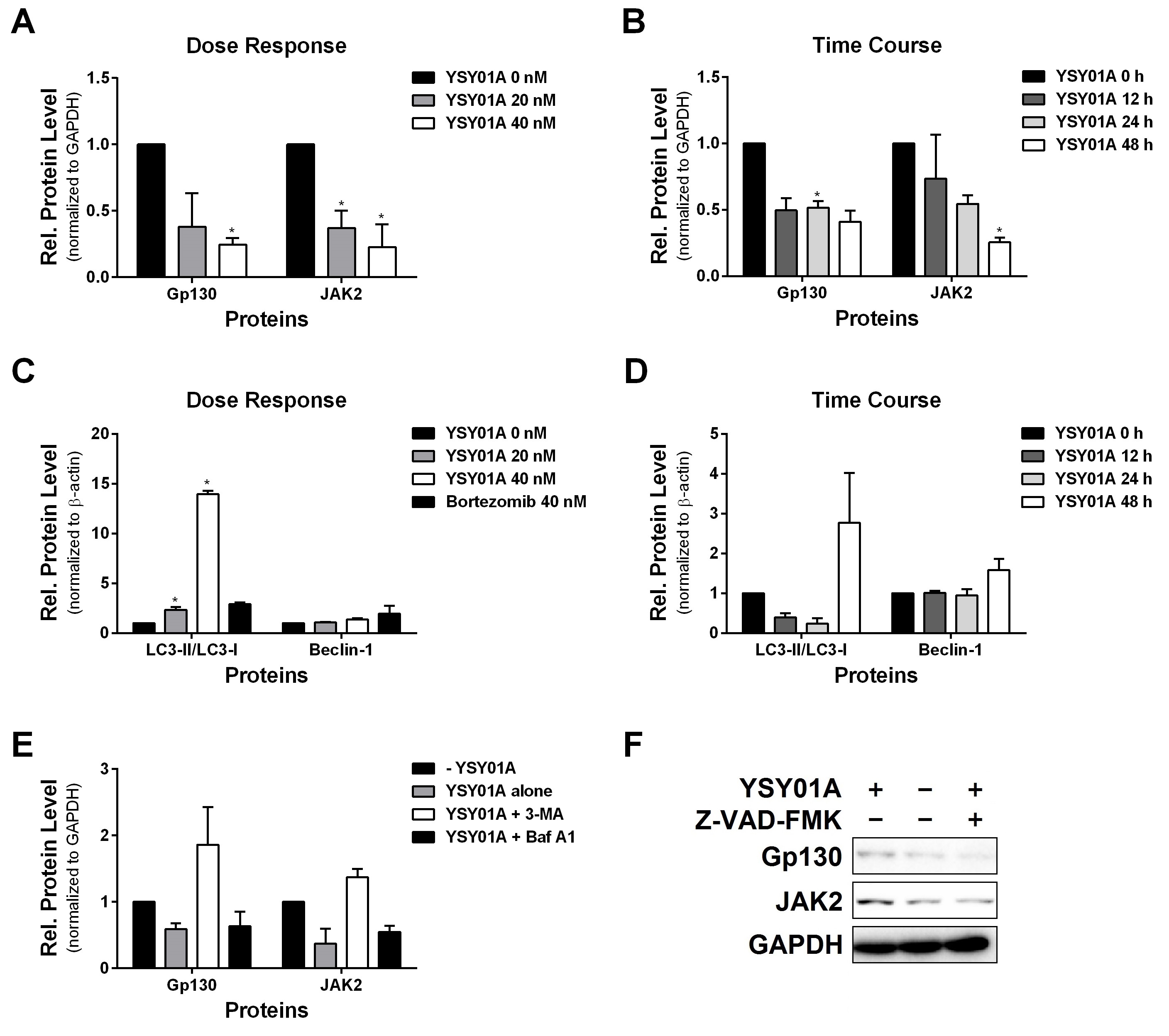

Supplement: Figure S3 — Expression levels of gp130 and JAK following treatment. Quantification of Figure 4 measured by the density of Western blot bands and normalized against the loading control. (A and C) A549 cells were treated with 0 (vehicle control), 20 or 40 nM YSY01A, or 40 nM bortizomib for 48 h. (B and D) A549 cells were treated with 40 nM YSY01A for 0, 12, 24, or 48 h. (E) In the presence of YSY01A treatment, A549 cells were co-treated with 5 mM 3-methyladenine (3-MA) or 100 nM Baflomycin A1 (Baf A1) for 48 h. (F) A549 cells were exposed to 40 nM YSY01A for 48 h in the absence or presence of Z-VAD-FMK, a pan-caspase inhibitor. (*p < 0.05, by Student's t-test as compared with vehicle control). [file Image3.JPEG]
